# Feed-Forward versus Feedback Inhibition in a Basic Olfactory Circuit

**DOI:** 10.1371/journal.pcbi.1004531

**Published:** 2015-10-12

**Authors:** Tiffany Kee, Pavel Sanda, Nitin Gupta, Mark Stopfer, Maxim Bazhenov

**Affiliations:** 1 Department of Cell Biology and Neuroscience, University of California, Riverside, Riverside, California, United States of America; 2 Department of Biological Sciences and Bioengineering, Indian Institute of Technology Kanpur, Kanpur, India; 3 US National Institutes of Health, National Institute of Child Health and Human Development, Bethesda, Maryland, United States of America; University of Chicago, UNITED STATES

## Abstract

Inhibitory interneurons play critical roles in shaping the firing patterns of principal neurons in many brain systems. Despite difference in the anatomy or functions of neuronal circuits containing inhibition, two basic motifs repeatedly emerge: feed-forward and feedback. In the locust, it was proposed that a subset of lateral horn interneurons (LHNs), provide feed-forward inhibition onto Kenyon cells (KCs) to maintain their sparse firing—a property critical for olfactory learning and memory. But recently it was established that a single inhibitory cell, the giant GABAergic neuron (GGN), is the main and perhaps sole source of inhibition in the mushroom body, and that inhibition from this cell is mediated by a feedback (FB) loop including KCs and the GGN. To clarify basic differences in the effects of feedback vs. feed-forward inhibition in circuit dynamics we here use a model of the locust olfactory system. We found both inhibitory motifs were able to maintain sparse KCs responses and provide optimal odor discrimination. However, we further found that only FB inhibition could create a phase response consistent with data recorded *in vivo*. These findings describe general rules for feed-forward versus feedback inhibition and suggest GGN is potentially capable of providing the primary source of inhibition to the KCs. A better understanding of how inhibitory motifs impact post-synaptic neuronal activity could be used to reveal unknown inhibitory structures within biological networks.

## Author Summary

Understanding how inhibitory neurons interact with excitatory neurons is critical for understanding the behaviors of neuronal networks. Here we address this question with simple but biologically relevant models based on the anatomy of the locust olfactory pathway. Two ubiquitous and basic inhibitory motifs were tested: feed-forward and feedback. Feed-forward inhibition typically occurs between different brain areas when excitatory neurons excite inhibitory cells, which then inhibit a group of postsynaptic excitatory neurons outside of the initializing excitatory neurons’ area. On the other hand, the feedback inhibitory motif requires a population of excitatory neurons to drive the inhibitory cells, which in turn inhibit the same population of excitatory cells. We found the type of the inhibitory motif determined the timing with which each group of cells fired action potentials in comparison to one another (relative timing). It also affected the range of inhibitory neurons’ activity, with the inhibitory neurons having a wider range in the feedback circuit than that in the feed-forward one. These results will allow predicting the type of the connectivity structure within unexplored biological circuits given only electrophysiological recordings.

## Introduction

**Fig 1:**
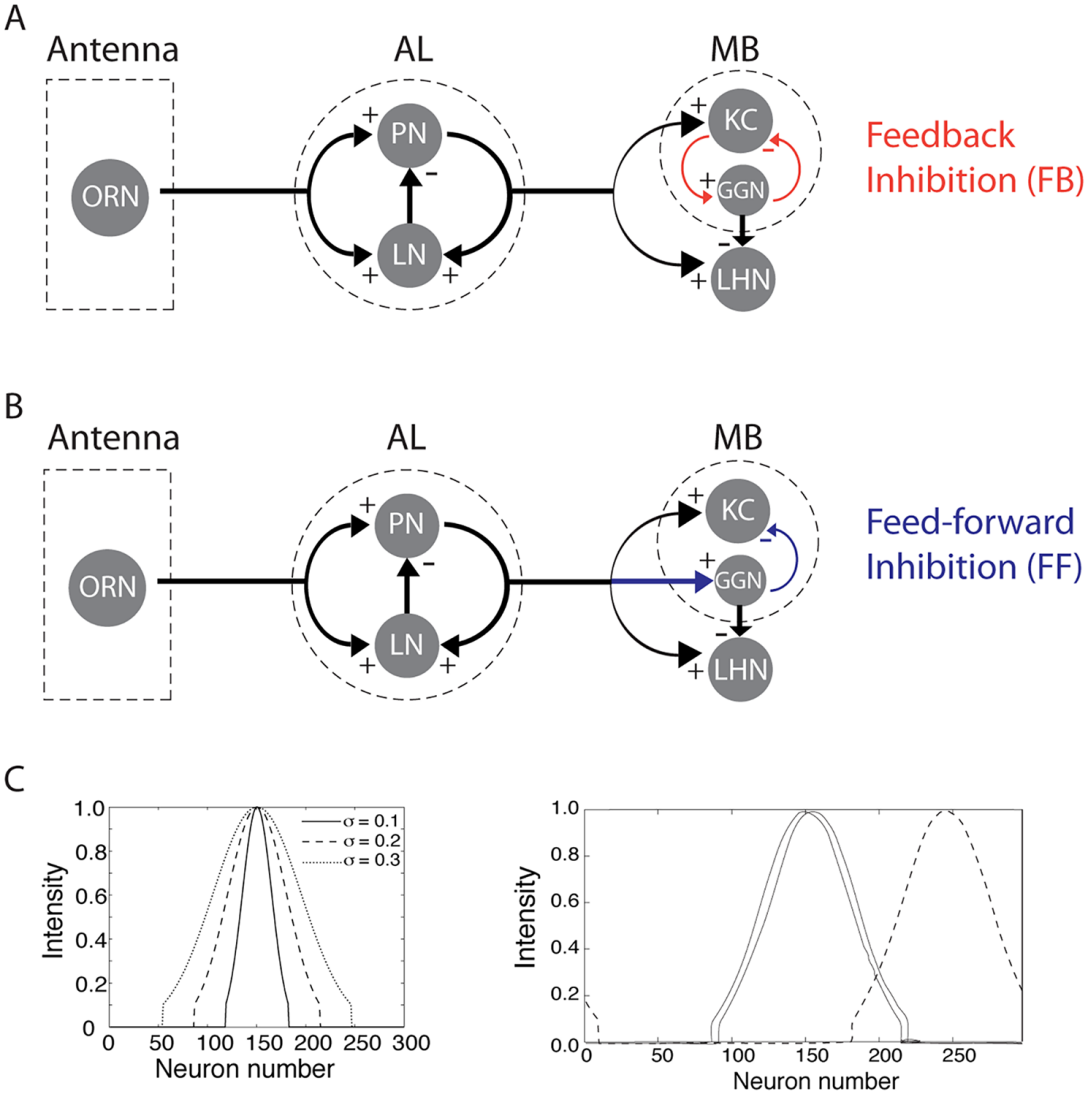
Schematic diagram of the models. (A) This circuit, based on results obtained *in vivo*, contains feedback inhibition between the KCs and GGN. (B) This hypothetical circuit contains feed-forward inhibition from the PNs to GGN inhibiting the KCs. (C) Left: Intensity of odor input received by each of the 300 PNs for representative odor concentrations (higher concentrations activate more PNs). Right: Different odors activate different sets of PNs; the two solid lines represent similar odors (activating largely overlapping subsets of PNs), and the dashed line represents a very different odor (activating a largely separate subset of PNs).

Inhibition is ubiquitous in invertebrate and vertebrate neural networks, and serves many different functions throughout the central nervous system. Within each neural network inhibition appears in various motifs, reflecting the connections between the excitatory and inhibitory neurons. Feed-forward (FF) and feedback (FB) are two very common, simple inhibitory network motifs [1–3]. FB (Fig 1A), or recurrent, inhibition requires a population of excitatory neurons to drive the inhibitory cell(s), which in turn inhibit(s) the same population of excitatory cells. FF inhibition (Fig 1B) typically occurs between different brain areas when excitatory neurons excite inhibitory cell(s), which then inhibit(s) a group of postsynaptic excitatory neurons outside of the initializing excitatory neurons’ area. While both types of inhibition can limit the firing of the postsynaptic neurons, some specific properties of these two basic inhibitory motifs are different. For example, because FF inhibition is controlled by upstream excitatory neurons, it is able to completely block action potentials in the post-synaptic excitatory neurons, and create relatively fast inhibition, setting the stage for precise temporal processing [4]. And FB inhibition is well suited to synchronize firing in populations of excitatory principal neurons [5].

Sparse stimulus representations can arise through either FF or FB inhibitory motifs [6]. FF and FB inhibition co-exists in the honeybee and mammal MB [7, 8]. In the vertebrate olfactory pathway FF inhibition in the cortex produces transient early-onset inhibition, while FB inhibition in the olfactory cortex produces late-onset inhibition [7]. Earlier reports suggested the existence of FF inhibition onto the Kenyon cells (KCs) in the mushroom body (MB), mediated by the lateral horn interneurons (LHIs), which themselves receive excitatory input from projection neurons (PNs) of the antennal lobe (AL) [9–11].

But more recent results in the fly and locust support the presence of FB inhibition in the MB and refute the existence of FF inhibition there: the recently characterized giant GABAergic neuron (GGN) now appears to receive excitatory input from all KCs and in turn inhibits all KCs, providing the main or sole source of inhibition in the MB [12–15]. FB inhibition creates longer windows for coincidence detection, but cannot entirely suppress the firing of excitatory population since the inhibition depends upon drive from the excitatory cells. Thus, such circuitry commonly generates oscillations with the degree of synchronization controlled by the strength of inhibition [5]. FB inhibition exists in the MB of fly, cockroach, locust, honeybee, and mammal [13, 14, 16, 17]. This FB inhibition has been tied to odor memory in many of these insects [13, 14, 16].

Sparse coding is thought to be important for learning and memory [18]. Sparse codes are based on a few stimulus-elicited spikes in a small subset of neurons within a large population of neurons. In many insects, sparse codes have been found in the KCs of the MB, a structure involved in memory and learning [13, 14, 19–22]. Sparse codes minimize inter-odor correlations and reduce interference between memory traces [9, 18]. When synaptic inhibition is blocked, in the fly and locust, by the GABA antagonist picrotoxin, the sparseness of the odor response in the MB decreases [9, 23], causing an increase in inter-odor response correlation, preventing insects from discriminating similar (but not dissimilar) odors [13, 24]. Inhibition in the MB changes with learning [25]. The FB inhibitory circuit also appears to be important for labile memory but not long-term memory [14].

To clarify the properties of FF and FB inhibition, we developed two models of the locust olfactory system. The first model implements FB inhibition that reflects our current understanding of the locust, in which KCs excite GGN, which, in turn, inhibits the same KCs (Fig 1A). The model implementing FF inhibition provides an alternative for comparison. (FF connectivity may exist in analogous circuits in other species: honeybees provide evidence for synapses between PNs and inhibitory fibers in the MB [8]). To allow direct comparison of the properties of FF and FB inhibitory motifs, in our FF model inhibition is also mediated by GGN: PNs from the antennal lobe excite GGN which then sends inhibition forward to the KCs in the mushroom body (Fig 1B). Our study revealed important differences between effects of FF and FB inhibition on circuit dynamics. Since some of these differences, such as timing of cell firing, can be evaluated given only electrophysiological recordings, our results provide a possible way to probe the connectivity structures of unexplored biological circuits.

## Materials and Methods

### Antennal lobe model

The locust contains about 850 PNs and about 300 LNs. Our model was scaled down to 300 PNs and 100 LNs with single compartments that included voltage- and Ca^2+^-dependent currents described by Hodgkin-Huxley kinetics [26]. Parameterization was done to minimize the number and complexity of ionic currents in each cell type and generate realistic (though simplified) firing profiles. No attempt was made to produce intrinsic resonant oscillations (pacemaker properties) in LNs or PNs because such properties have never been observed in locust LNs or PNs [27, 28]. The model was constrained to produce population oscillations (LFP) in the AL and cellular responses as observed *in vivo* [5].

### Membrane potentials: PN and LN membrane potential equations [26]

$$\begin{array}{l} C_{m}\frac{dV_{PN}}{dt}=-g_{L}\left(V_{PN}-E_{L}\right)-I_{Na}-I_{K}-I_{A}-g_{KL}\left(V_{PN}-E_{KL}\right)-I_{GABA_{A}}-I_{nACh}-I_{stim}\\ C_{m}\frac{dV_{LN}}{dt}=-g_{L}\left(V_{LN}-E_{L}\right)-I_{Ca}-I_{K(Ca)}-I_{K}-g_{KL}\left(V_{LN}-E_{KL}\right)-I_{GABA_{A}}-I_{nACh}-I_{stim} \end{array}$$

The LN passive parameters are: C_m_ = 1 μF, g_L_ = 0.15 μS, g_KL_ = 0.02 μS, E_L_ = -50 mV, and E_KL_ = -95 mV. The PN passive parameters are the same as LN except: E_L_ = -55 mV, and g_KL_ = 0.05 μS. An external DC input was introduced to each neuron through I_stim_.

Intrinsic currents are described by equations:

Sodium current (I_Na_) [29]

$$I_{Na}=g_{Na}m^{3}h\left(V-E_{Na}\right),$$

where the conductance in PNs is g_Na_ = 7.15 μS, and E_Na_ = 50mV. The gating variable satisfies the equations:

$$\begin{array}{l} \frac{dm}{dt}=-\frac{1}{\tau_{m}}\left(m-m_{\infty }\left(V\right)\right)\\ \frac{dh}{dt}=-\frac{1}{\tau_{h}}\left(h-h_{\infty }\left(V\right)\right) \end{array}$$

The steady state values of the gating variables are given by:

$$\begin{array}{l} m_{\infty }\left(V\right)=\frac{1}{1+\text{exp}\left(-\frac{V+20}{6.5}\right)}\\ h_{\infty }\left(V\right)=\frac{1}{1+\text{exp}\left(\frac{V+25}{12}\right)} \end{array}$$

The time constants are:

$$\begin{array}{l} \tau_{m}=1.5\\ \tau_{h}=0.3\text{exp}\left(\frac{V-40}{13}\right)+0.002\text{exp}\left(-\frac{V-60}{29}\right) \end{array}$$

Fast potassium current (I_K_)[29]

$$I_{K}=g_{K}n^{4}\left(V-E_{K}\right),$$

where the conductance in LNs is g_K_ = 10 μS and E_K_ = -95 mV. In the PNs g_K_ = 1.43 μS and E_K_ = -95 m. The equation for the gating variable n is given by:

$$\frac{dn}{dt}=-\frac{1}{\tau_{n}}\left(n-n_{\infty }\left(V\right)\right)$$

where the steady state value, n_∞_, and the time constant, τ_n_, are nonlinear functions of V and given by,

$$\begin{array}{l} n_{\infty }=\frac{\alpha_{n}}{\left(\alpha_{n}+\beta_{n}\right)\phi }\\ \tau_{n}=\frac{1}{\left(\alpha_{n}+\beta_{n}\right)\phi } \end{array}$$

The variable ϕ depends on the temperature and is given by $\phi =3^{\left(\frac{22-26}{10}\right)}$ at 26°C.

$$\begin{array}{l} \alpha_{n}=0.02\frac{\left(15-V_{shift}\right)}{\text{exp}\left(\frac{10-V_{shift}}{40}\right)}\\ \beta_{n}=0.5\text{exp}\left(\frac{10-V_{shift}}{40}\right) \end{array}$$

Ca^2+^ current (I_Ca_)

$$I_{Ca}=g_{Ca}m^{2}h\left(V-E_{Ca}\right)$$

where g_Ca_ = 2 μS and E_Ca_ = 140 mV. The gating variables satisfy the equations,

$$\begin{array}{l} \frac{dm}{dt}=-\frac{1}{\tau_{m}}\left(m-m_{\infty }\left(V\right)\right)\\ \frac{dh}{dt}=-\frac{1}{\tau_{h}}\left(h-h_{\infty }\left(V\right)\right) \end{array}$$

The steady state values of the gating variables are given by,

$$\begin{array}{l} m_{\infty }\left(V\right)=\frac{1}{1+\text{exp}\left(-\frac{V+20}{6.5}\right)}\\ h_{\infty }\left(V\right)=\frac{1}{1+\text{exp}\left(\frac{V+25}{12}\right)} \end{array}$$

The time constants are τ_m_ = 1.5 and

$$\tau_{h}=0.3\text{exp}\left(\frac{V-40}{13}\right)+0.002\text{exp}\left(-\frac{V-60}{29}\right)$$

Calcium-dependent potassium current (I_K(Ca)_)

$$I_{KCa}=g_{KCa}m^{2}h\left(V-E_{KCa}\right)$$

where g_KCa_ = 0.3 μS and E_KCa_ = -90 mV. The gating variable satisfies the equation:

$$\frac{dm}{dt}=-\frac{1}{\tau_{m}+\tau_{x}}\left(m-m_{\infty }\left(V\right)\right)$$

While,

$$\begin{array}{l} m_{\infty }\left(V\right)=\frac{\left[Ca^{2+}\right]}{\left[Ca^{2+}\right]+2}\\ \tau_{m}=\frac{100}{\left[Ca^{2+}\right]+2} \end{array}$$

and τ_x_ is obtained from a uniform distribution extending from -0.02 to 0.01. The calcium concentration satisfies a simple first order equation:

$$\frac{d\left[Ca^{2+}\right]}{dt}=-AI_{Ca}-\frac{\left(\left[Ca^{2+}\right]-{\left[Ca^{2+}\right]}_{\infty }\right)}{\tau }$$

where [Ca^2+^]_∞_ = 2.4 * 10^−4^ mM is the equilibrium of intracellular Ca^2+^ concentration, A = 5.2 * 10^−4^ mM * cm^2^/(ms * μA) and τ = 5 ms.

Transient potassium A current (I_A_)

$$I_{A}=g_{A}m^{4}h\left(V-E_{A}\right)$$

where g_A_ = 10 μS, and E_A_ = -95 mV. The steady state values of the gating variables are given by:

$$\begin{array}{l} m_{\infty }\left(V\right)=\frac{1}{1+\text{exp}\left(-\frac{V+60}{8.5}\right)}\\ h_{\infty }\left(V\right)=\frac{1}{1+\text{exp}\left(\frac{V+78}{6}\right)} \end{array}$$

The time constants were given by:

$$\tau_{m}=\frac{0.25}{\left[\text{exp}\left(\frac{V+35.8}{19.7}\right)+\text{exp}\left(-\frac{V+79.7}{12.7}\right)+0.09\right]}$$

$$\tau_{h}=\frac{0.25}{\left[\text{exp}\left(\frac{V+46}{5}\right)+\text{exp}\left(-\frac{V+238}{37.5}\right)\right]}$$

if *V* < −63 *mV* and *τh* = 4.8 *if V* ≥ -63 *mV*.

#### Synaptic Currents

Fast GABA and nicotinic cholinergic synaptic currents to LNs and PNs [28] are modeled by first-order activation schemes (see review in [30]). Fast GABA and cholinergic synaptic currents are given by:

$$I_{syn}=g_{syn}\left[O\right]\left(V-E_{syn}\right)$$

where the reversal potential is E_nACh_ = 0 mV for cholinergic receptors and E_GABAA_ = -70 mV for fast GABA receptors. The fraction of open channels [O] is calculated according to the equation

$$\frac{d\left[O\right]}{dt}=\alpha \left(1-\left[O\right]\right)\left[T\right]-\beta \left[O\right]$$

For cholinergic synapses

$$\left[T\right]=A\Theta \left(t_{0}-t_{\text{max}}-t\right)\Theta \left(t-t_{0}\right)$$

and for GABAergic synapses

$$\left[T\right]=\frac{1}{1+\text{exp}\left(-\frac{V\left(t\right)-V_{0}}{\sigma }\right)}$$

Θ is the Heaviside step function [31], t_0_ is the time of receptor activation, A = 0.5, t_max_ = 0.3 ms, V_0_ = -20 mV, and σ = 1.5. The rate constants, α and β, were α = 10 ms^-1^ and β = 0.16 ms^-1^ for GABA synapses and α = 10 ms^-1^ and β = 0.2 ms^-1^ for cholinergic synapses. The peak synaptic conductances were set to g_GABAA_ = 4 * 10^−4^ between LNs, g_GABAA_ = 2 * 10^−4^ from LNs to PNs and g_ACh_ = 5 * 10^−4^ μS from PNs to LNs.

#### Network geometry

In the locust AL, LNs are synaptically connected to other LNs and to PNs [32]. Both LNs and PNs receive direct synaptic input from olfactory receptor neurons [28].

All network interconnections were random with 0.5 probability, suitable for our scaled-down network. Some of the intrinsic parameters of the neurons in the network were initialized with random variability to ensure robust results. Also, small-amplitude current in the form of Gaussian noise (σ ≈ 10%) was introduced to each cell to achieve realistic, random and independent membrane potential fluctuations.

### Input to the AL model

Similar to [10], odor identity was determined by the identities of PNs activated by the input, modeled as a current pulse injected to each cell that was activated by a given odor. Odorant similarity was determined by the spatial overlap between two odor inputs. Odor concentration was modeled as the relative size of LNs and PNs populations activated by input (Fig 1C). The lowest concentration activated ~10%, and the highest concentration activated ~80%, of the LNs and PNs. Each concentration in between activated an additional ~10% more neurons than the previous concentration. In the AL, the actual number of LNs and PNs activated by an odor is estimated to be smaller, ~10–20% [28], but the population size here was chosen to scale properly with our model. Stimuli were modeled by current pulses with a rise time constant of 100ms and decay constant of 200ms. The current used for each pulse was calculated as the total synaptic current produced by N Poisson distributed spike trains (each with average spike rate μ) arriving at N-independent excitatory synapses. Each glomerulus in the locust AL receives between 100 and 200 axons from olfactory receptor neurons [28]; thus N was set to 200 and μ was varied between 50 and 150 Hz. In this case, random current fluctuations were 5%-10% of its amplitude; lower μ produced slightly higher current fluctuations and vice versa. We used μ = 100 Hz for the simulations presented here to match the membrane potential fluctuations observed in postsynaptic PNs *in vivo* [33].

### Mushroom body, lateral horn, and GGN model

To model the large population (15,000) of neurons in the MB while maintaining practical run times, we used map-based model neurons [34, 35] for the KCs. Similar models have been used for LH, and GGN to facilitate synaptic interactions between these cell populations. Since there are no feedback projections from downstream populations back to the AL, simulations of the AL were run independently using HH models and PN spike trains were saved and then used as input for MB-LH-GGN simulations.

Map-based KC and LHN models are described by the second order map [34–36]:

$$\begin{array}{l} x_{n+1}=f_{\alpha }\left(x_{n},x_{n-1},y_{n}+\beta_{n}\right)\\ y_{n-1}=y_{n}-\mu \left(1+x_{n}\right)+\mu \sigma +\mu \sigma_{n} \end{array}$$

Here, *x* represents membrane voltage, and *y* describes slow cellular dynamics such as spike adaptation. β_n_ and σ_n_ describe external influences on the system, such as synaptic inputs (I_n_^syn^); α, σ, and μ are parameters that affect spiking patterns of the model [34, 35]. Here we selected values for KCs and LHNs as following: μ = 0.0005, σ = 0.06, β = 0.03. For KCs the parameter μ (affecting slow dynamics of the membrane) was drawn for each neuron from uniform distribution from the interval of 0.0012±0.00068 for KCs.

Excitatory AMPA synapse was described as following [35]

$$I_{n+1}^{syn}=\gamma I_{n}^{syn}+\{\begin{array}{c} g_{syn}\left(x_{n}^{post}-x_{rp}\right),\;spike_{pre},\\ 0,\;otherwise. \end{array}$$

Since GGN is known to produce non-spiking response [11], we modeled it using the following equations:

$$\begin{array}{l} x_{n+1}=\alpha \left(x_{n}-y_{n}\right)\\ y_{n+1}=y_{n}+\mu \left(1+x_{n}\right)-\mu \left(\sigma +\sigma_{n}\right) \end{array}$$

While GGN synaptic strength was different for two inhibitory motif models, the inhibition it produces was comparable between the models. Indeed, it was tuned to produce spike counts within the same physiological range (discussed below) in both models across the entire tested range of concentrations. We tested different connectivity probabilities and found no substantial differences in network dynamics as long as the strengths of individual synapses were scaled accordingly. We selected the GGN variables as follows: α = 0.8, μ = 0.005, σ = −0.5, σ_e_ = 1.0, σ_n_ = σ_e_ * I.

Inhibitory GABAergic synapse was described as following [35]

$$I_{n+1}^{syn}=\gamma I_{n}^{syn}+\{\begin{array}{c} \left(1/1+e^{\left(-x+1.5\right)/1.5}\right)g_{syn}\left(x_{n}^{post}-x_{rp}\right),\;x_{n}>-1.4,\\ 0,\;otherwise, \end{array}$$

**Geometry**. We modeled 15,000 KCs. The connectivity from PNs to KCs was random and had a probability of 0.33 and strength of 0.00066. The model included 40 spiking LHNs. The connectivity from PNs to LHNs was random and had a probability of 0.7 and strength of 0.007.

#### Feed-forward network geometry (Fig 1B)

PNs excite GGN through AMPA synapses with synaptic strength 0.02. In this model the KCs do not excite GGN but do receive inhibition through the GGN’s GABAergic synapses with a synaptic strength of 0.000035. GGN makes GABAergic synapses onto LHNs with a synaptic strength of 0.00027.

#### Feedback network geometry (Fig 1A)

PNs do not excite the GGN. KCs excite the GGN with AMPA synapses with synaptic strength of 0.5. KCs receive inhibition through the GGN’s GABAergic synapses with a synaptic strength of 0.00004. GGN makes GABAergic synapses to LHNs with a synaptic strength of 0.00045.

### Data analysis

#### Cohen’s d

This measure of effect size is calculated with the means and standard deviations of the two groups of interest:

$$d=\frac{M_{1}-M_{2}}{\sqrt{\frac{SD_{1}^{2}+SD_{2}^{2}}{2}}}$$

To measure the effective size of GGN output conductance for each concentration we took the average conductance across the entire odor presentation, and averaged across trials yielding a GGN output conductance for each odor and concentration. For the concentration-specific analysis we calculated the mean, standard deviation and Cohen’s d by combining all the odors for each concentration. For the model specific calculation we calculated the mean, standard deviation, and Cohen’s d by combining all the odors and concentrations for each model.

#### Population sparseness

Population sparseness was used to measure how sparsely the KC population responded to each odor [37]:

$$S_{p}=\frac{1-\frac{{\left(\sum_{j}^{N}r_{j}/N\right)}^{2}}{\sum_{j}^{N}r_{j}^{2}/N}}{1-\frac{1}{N}}$$

*N* is the number of neurons (15000 KCs), *r*_*j*_ is the response of neuron *j*. S_p_ = 1 means that no cells respond with spikes to a stimulus; S_p_ = 0 means that every cell responds with spikes to a stimulus. The sparseness value is most influenced by the fraction of cells within the population that responds to the odor.

#### Odor radius

Odor representation within each cell population is described in *K*-dimensional space (*K* = 15,000 for KCs, *K* = 300 for PNs and *K* = 100 for LHNs); the spike count of each neuron of a given type over the duration of the odor stimulation is represented in one dimension so that a single point in this space represents the response of the entire population to a stimulus, and repeated trials create a “cloud” of such points. This cloud is a representation of an odor in the coding space, and we define its radius as the average of distances from the center to each trial’s point. This approach was used to represent odor in KC, PN or LHI spaces.

#### Odor distance

The average across all the trials of a given odor was defined as a center of the cloud. To determine differences in response to two odors, we calculated the Euclidean distance between the averages (centers) of responses elicited by the two odors.

#### Odor classification error

Classification error was calculated between two odors: A and B [38]. After each odor trial and its odor center are calculated (see above), an error is scored when a trial of odor A is closer (Euclidean distance) to the center of the cloud representing the other odor (B) than to its own odor cloud center (A). The classification error graphs show the percentage of trials including errors.

#### Cell phase

PN voltages were averaged and low-pass filtered with frequency cutoffs at 50 Hz to simulate local field potentials (LFPs). PN, KC, and LHN spike times were converted to phases with respect to the LFP. The positive peaks of the field potential were assigned to phase 0 or 2π, based on the nearest minima, which were assigned +π or –π, respectively. The phase of each cell spike was calculated relative to the nearest peak of the field potential according to the equation [28]:

$$\Phi_{Cspike}=\left(\frac{t_{Cspike}-t_{lastLFPpeak}}{t_{nextLFPpeak}-t_{lastLFPpeak}}\right)2\pi$$

C_spike_ represents the spike of a specific cell type: PNs, KCs, or LHNs. For GGN C_spike_ is the peak activity within oscillation cycle; if the GGN membrane potential does not peak during the oscillation, that oscillation is skipped and calculations continue through the rest of the oscillations.

### Simplified model

A simplified model was created using map-based neurons with the same parameter values as described above. This model includes a single KC, LHN, and GGN. The neurons in the model received a sine wave to represent input from AL:

y(t)=Asin(wt)

A is the amplitude of the sine wave and w is the angular frequency (2πf). A = 0.4, w = 0.076.

Feedback parameters: g_ampa_GGNKC_ = 0.001, g_gaba_KCGGN_ = 0.0001–0.0004, g_gaba_LHGGN_ = 0.0001–0.0004, g_ampa_KCPN_ = 0.3, g_ampa_LHPN_ = 1.5, and g_ampa_GGNPN_ = 3.0. Feedforward parameters: g_ampa_GGNKC_ = 0.0, g_gaba_KCGGN_ = 0.0001–0.0004, g_gaba_LHGGN_ = 0.0001–0.0004, g_ampa_KCPN_ = 0.3, g_ampa_LHPN_ = 1.5, and g_ampa_GGNPN_ = 3.0.

## Results

### Inhibitory motifs and sparseness of the population response

**Fig 2:**
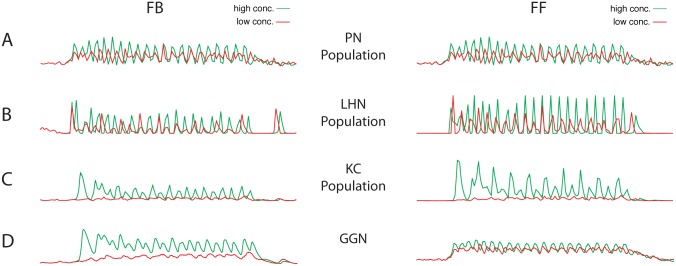
Population activity elicited by different odor concentrations, low (red) and high (green), in FB and FF models. Activity plots show the number of population action potentials except for the nonspiking GGN, which shows graded intracellular membrane potential. (A) PN population. (B) LHN population. (C) KC population. (D) GGN.

Anatomical evidence suggests that GGN inhibits both the KCs in the mushroom body (MB) and the LHNs in the lateral horn (LH), yet KCs fire sparsely while LHNs respond to odors with dense spiking [9, 12]. Thus, it is important to determine whether the influence of GGN is consistent with the disparity in activity seen between the MB and LH. In the following we compare results obtained from network models of the insect olfactory system based on FB and FF inhibition (Fig 1A and 1B). In the FB model excitatory input from KCs drove GGN, which, in turn, sent inhibition back to KCs. In the FF model PNs from the AL drove GGN, which in turn inhibited the KCs. While recent anatomical evidence from the well-studied locust olfactory system supports the FB model [12], both FB and FF mechanisms have been proposed and may likely exist in insect olfactory systems. Thus, our motivation was to use this system to explore general properties in circuit dynamics arising in the two models reflecting motifs commonly found in biological networks [6]. In both FF and FB models all cell populations showed oscillatory behavior, since the pacemaker for the whole circuit resides in the AL and consists of interactions between PNs and LNs (Fig 2) [5]. In both models GGN responds to odors with non-spiking but oscillatory synchronized responses (Fig 2D), as has been shown empirically [12, 19].

**Fig 3:**
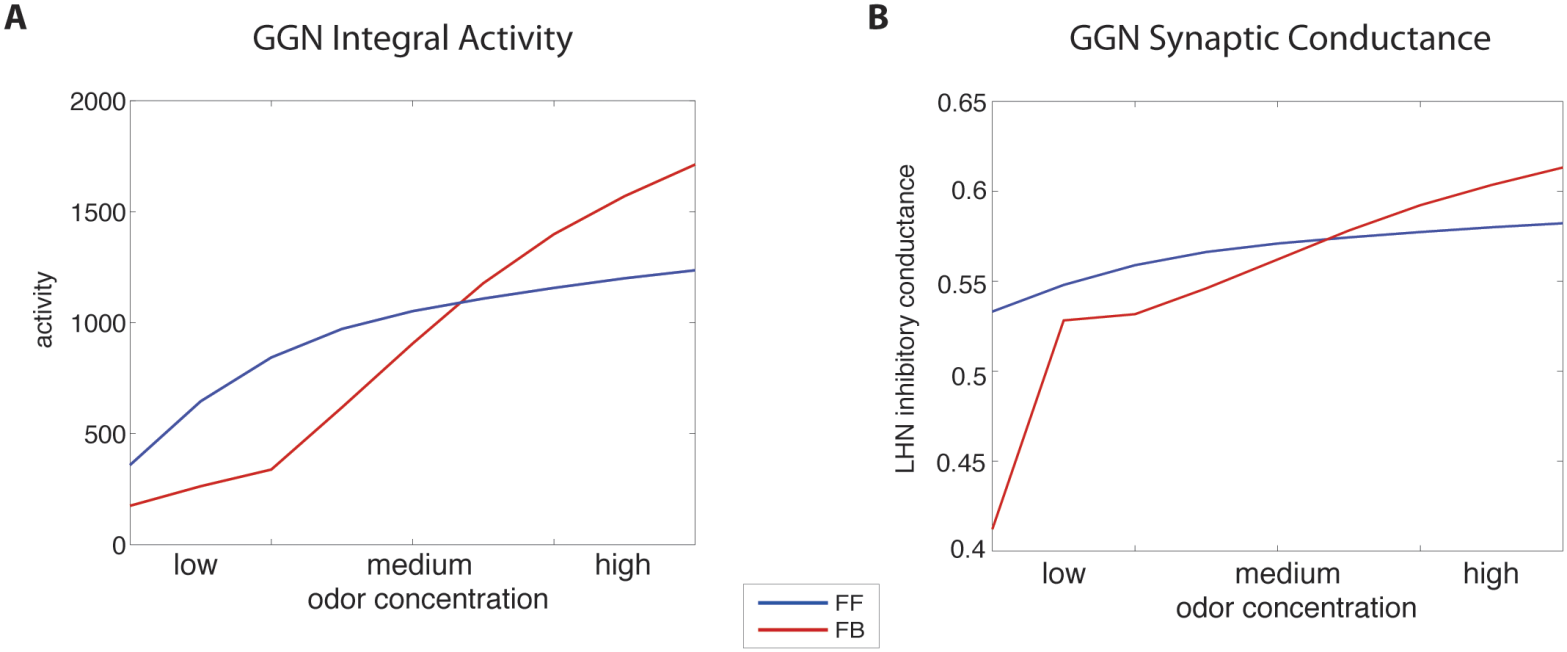
GGN activity and its inhibitory input to the LHNs across a range of concentrations. Colors indicate the type of the motif: feed-forward (FF) inhibition (blue) and feedback (FB) inhibition (red). (A) The activity of GGN calculated by integrating GGN’s membrane depolarization over the stimulus duration. (B) Inhibitory input to the LHNs from GGN for different odor concentrations.

GGN provides inhibitory input to both KCs and LHNs and is the focal point for both inhibitory motifs. We found that regardless of the model, GGN increased its activity as odor concentration increased (Fig 3A). Our models showed that at low odor concentrations GGN was more excitable (estimated by calculating the integral of activity over time) in the FF model than in FB one, but this reversed at higher odor concentrations (Fig 3A). This result revealed distinct motif-specific responses, as FF inhibition produces a more responsive GGN at low concentrations, and FB model produces a more active GGN at high odor concentrations. Overall, in the FB model GGN activity spanned a broader range of responses, compared to the FF model. Although synaptic inhibition was significantly different for each specific concentration (Cohen’s d > 1.5) (Fig 3A), there was not a significant difference between FF (M = -0.05, SD = 0.001) and FB (M = -0.05, SD = 0.003) models across the entire range of odor concentrations (Cohen’s d < 0.005).

Fig 3B shows the input into the LHNs from the GGN for the two models. The inhibitory input follows a similar profile to that of the GGN activity, with main differences emerging at very low concentrations where FF model delivered significantly stronger inhibition than the FB model. Thus, the strength of GGN inhibition was larger in the FF model vs the FB model for low odor concentrations, and it was the opposite for high odor concentrations. This also shows that one cannot tune both models to be precisely equivalent across the entire range of odor concentrations. These results highlight inherent differences between the two inhibitory motifs and contribute to the distinct properties of the two models, as we report below.

**Fig 4:**
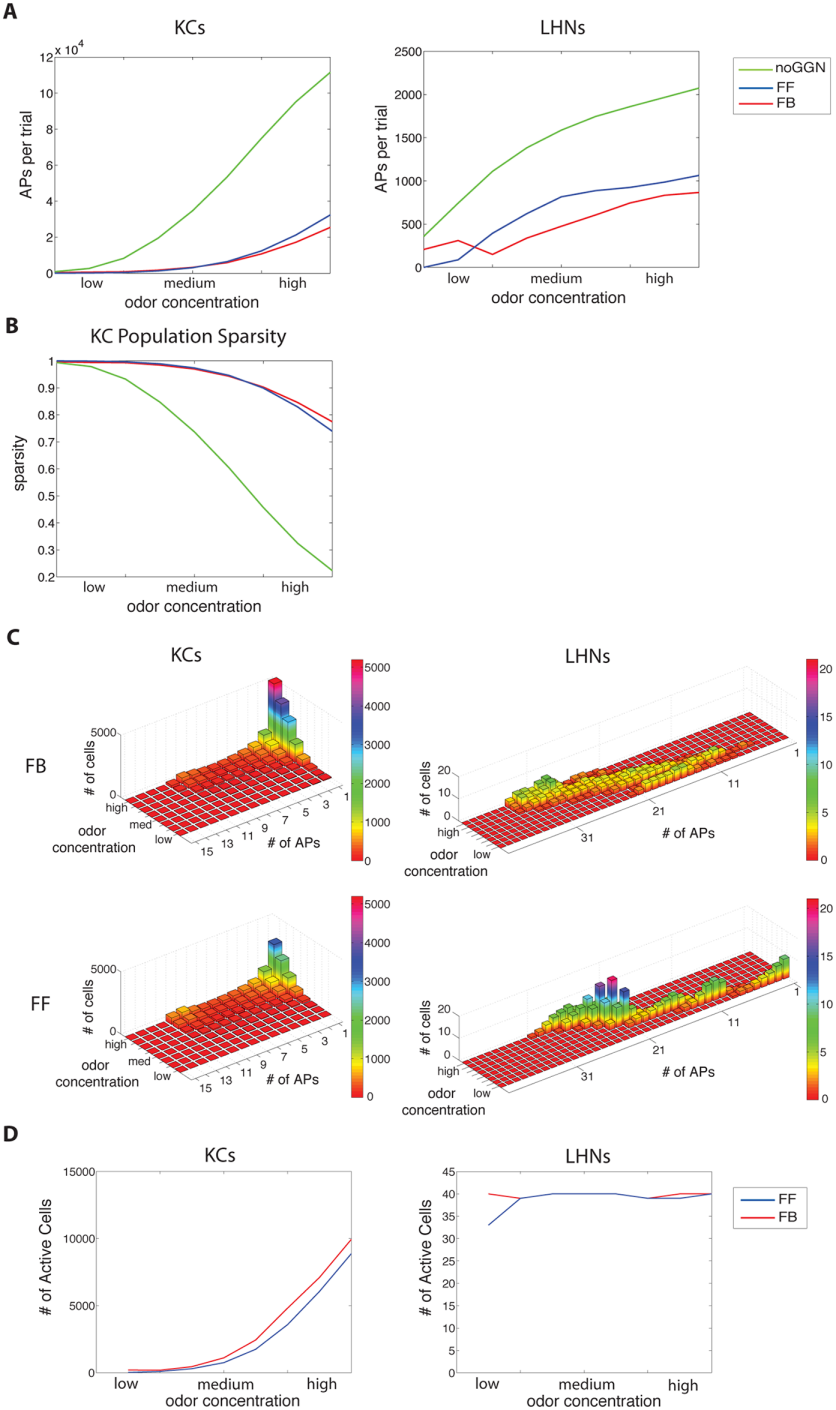
Network activity and response sparseness generated by different inhibitory motifs. Colors indicate these motifs: no giant GABAergic neuron inhibition (noGGN) (green), feed-forward (FF) inhibition (blue), and feedback (FB) inhibition (red). (A) The average numbers of action potentials (APs) per trial for Kenyon cells (KCs). The average numbers of action potentials (APs) per trial for lateral horn neurons (LHNs). (B) The sparseness of odor representation across all Kenyon cells. (C) Frequency distribution of KC and LHN response intensity (total number of spikes elicited by a 1-s odor presentation) for different odor concentrations. (D) The number of cells generating an action potential in response to stimuli. Very high numbers of APs and unrealistically active KCs elicited by “high” odor concentrations suggest that the physiological range usually explored *in vivo* corresponds to “low-medium” range of the model.

We next measured population activity of KCs and LHNs. Inhibition from GGN regulated action potentials produced by KCs (Fig 4A, left). Most KCs were silent, regardless of the inhibitory motif. Inhibition not only decreased the number of overall action potentials but also decreased the number of cells responding to the odor (Fig 4B). And, as expected [10], population sparseness was much greater when inhibition was included in the models. Responsive KCs typically fired a single action potential (Fig 4C). Both FF (blue line) and FB (red line) models limited the number of action potentials elicited by all concentrations of odor presentations (Fig 4D, left). However, as odor concentration increased, the FF model allowed greater increases in the total number of action potentials. By contrast, the FB model kept the number of action potentials constant, but allowed the number of cells responding to an odor to increase along with the concentration. This was caused by differences in inhibitory input generated by the GGN response. Quantitatively, across the full range of odor concentrations and all odors in the FF model, 18% of KCs responded with spiking; the average response was four spikes. In the FB model, 21% of active KCs fired 3 action potentials. Considering only the center of the range of odor concentrations (conc. 0.2 and 0.25), 8.5% of active KCs in the FF model fired an average of 3.8 action potentials; 11.5% of active KCs in the FB model fired an average of 2.6 action potentials. We conclude that both FF and FB inhibition can constrain the MB to respond sparsely to odors, thus fulfilling a requirement for efficient formatting for memory storage.

Results recorded *in vivo* show KCs respond sparsely while LHNs respond to every odor with multiple spikes [12]. Compared to KCs, model LHNs responded with greater levels of activity to odors, despite inhibitory input from GGN. Both FF (blue) and FB (red) inhibition greatly limited the number of LHN action potentials, compared to the model with no inhibition (green) (Fig 4A right). In agreement with experimental results [12], a large fraction of modeled LHNs responded to each odor. Specifically, all LHNs responded to every odor, except for the lowest odor concentrations when 17.5% of LHNs did not respond in the FF condition (Fig 4D, right). As odor concentration increased both FF and FB motifs produced more action potentials in LHNs (Fig 4C, right). However, the two models generated different response profiles: FF inhibition produced a skewed action potential histogram for each concentration with most LHNs firing the fewest action potentials and a few cells firing the most, whereas FB inhibition produced a symmetrical LHN action potential histogram (Fig 4C). Regardless of the different action potential profiles, both inhibitory motifs supported the firing of LHNs in response to each odor with similar total numbers of action potentials.

### Odor classification error by KCs

**Fig 5:**
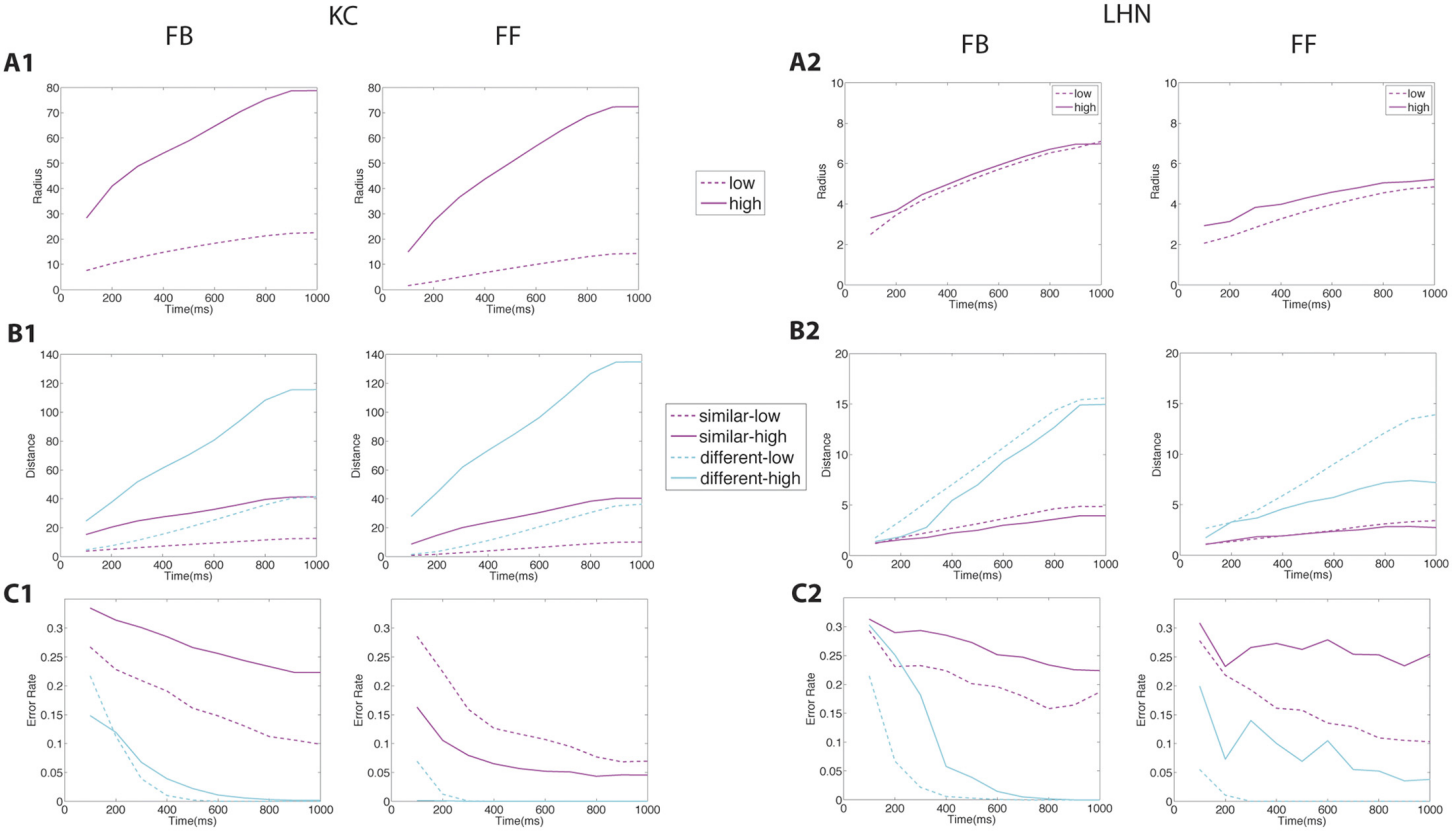
Effect of integration time on odor classification. For each motif (FB or FF) are shown: similar odors of low concentration (dotted magenta); similar odors of high concentration (magenta); different odors of low concentration (dotted blue); different odors of high concentration (blue). (A) The average radius of the “cloud” representing multiple trials for high and low odor concentrations. (A1) KCs. (A2) LHNs. (B) Euclidean distance between centers of “clouds” representing multiple trials of two odors. (C) Classification error across multiple trials with two odors.

*In vivo*, the responses of KCs can be used to effectively classify the odors that elicit the responses; that is, KCs have been shown to contain information about odors. To evaluate classification success of the responses of model KCs across a range of odor trials including realistic levels of noise, KCs spikes were counted within a time window beginning and ending with the odor presentation (1000 ms). Odor representations by the KC population were described with one dimension for each KC (thus, with a 15,000 dimension space). A single point in this space represents the response of the population to an individual odor trial, and repeated trials generate a “cloud” of such points. To quantify response variability we defined the cloud radius as an average distance from its center (mean point, center of mass [38]) to each trial’s point. The mean radius for each odor increased from low to high with odor concentration (Fig 5A1). It also increased when time windows for including responsive spikes were extended. Overall, the FF model produced smaller size clouds than the FB model; thus, the FF model generated responses more resistant to noise.

In our model, odor similarity is defined by the similarity (the amount of overlap) of the inputs to the antennal lobe (see Methods section). Thus, we could define odorants as similar or different, and could calculate Euclidean distances in the coding space of those odorants. In the following we considered two sets of odors: similar (99% overlap) and different (80% overlap). Since each odor stimulus was represented by repeated trials with added noise, we could calculate the distance between the centers of the odor response clouds in KCs as defined above. We found that, in KCs, the distance between responses to two odors of the same concentration, regardless of odor similarity, was nearly the same for FF and FB models. One exception was that different odors presented at high concentrations elicited larger distance measures in the FF than FB model (solid blue line Fig 5B).

To calculate odor classification success we took the previously described KC odor response clouds and calculated whether a trial was closer to the mean (center of mass) of its own odor cloud or closer to that of another test odor. If the trial was closer to the mean of the other odor cloud, the response was marked as an error; the fraction of odor trials with errors defined the error rate. We found that the FF model yielded slightly fewer odor classification errors than the FB model for all odor-concentration pairs (Fig 5C1). The smaller radius of the cloud representing odor responses (meaning fewer cells responding with more action potentials) in the FF model was primarily responsible for this disparity. Thus, under our test conditions, the FF inhibition model achieved slightly better discrimination between similar odors at the level of KCs.

### Odor representation by LHNs in FF vs FB models

Following the approach described above for KCs, odor representations by the LHNs were described in forty-dimensional space, one dimension for each LHN in the model. To determine the effect of stimulus duration on odor classification success, we varied the duration of the time window within which we summed numbers of spikes elicited by each odor presentation. We then compared the size of the cloud representing multiple trials of the same odor, measuring the distance between centers of two clouds and classification success, as described above. The average radius of a cloud representing responses of LHN over repeated trials remained remarkably constant across odor concentrations. In contrast, the radius of the cloud increased as lengthier odor presentation time windows were included in the analysis (Fig 5A2). We found that the FF inhibition model produced slightly smaller LHN response odor clouds, indicating more reliable responses. Although the Euclidean distance between responses to odors was generally similar for both FF and FB inhibitory motifs, small differences arose when the similarity of the odors we compared was varied (Fig 5B): in the FB model, different odors at high concentrations elicited better separated responses in LHNs than the FF model.

Odor classification success for LHN responses was also similar in FF and FB inhibitory motifs (Fig 5C). In all cases, similar odors were more difficult to classify than different odors, and high concentrations were more difficult to classify than low concentrations. The FB model yielded greater odor classification success for high odor concentrations, and the FF model had greater odor classification success for low odor concentrations. We concluded that, in the LHNs, a small cell population with densely spiking cells, FB inhibition was more effective for odor classification for high concentrations and FF inhibition was more effective for low odor concentrations.

### Phase response analysis

**Fig 6:**
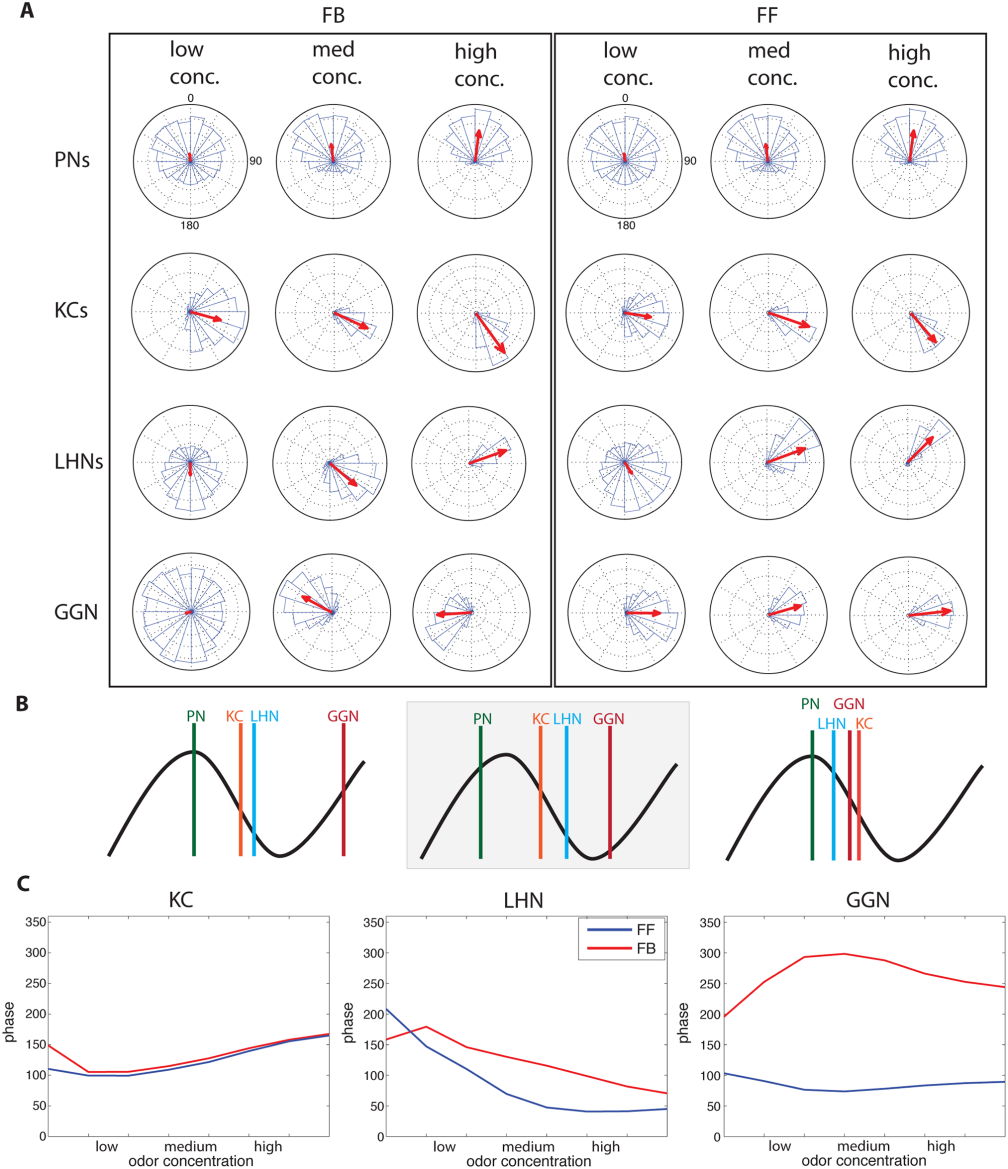
Phase locking across odor concentrations. (A) Circular phase graphs; length of the red arrow indicates the strength of phase locking; direction of the arrow indicated mean spike phase with respect to the field potential (peak of average activity in PNs, defined as zero phase). (Left) Feedback motif, PNs (top), KCs (middle), and LHNs (bottom), across low (left) and high (right) odor concentration. (Right) Feed-forward motif. (B) Schematic diagram comparing preferred firing phase of different cell types in FB model (left), FF model (right) and recordings made *in vivo* from locust (middle). (C) Phase locking across odor concentrations. (Left) KCs show stable phase locking (minimal phase change across concentrations) with more stability in the FB condition (red) than the FF condition (blue). (Middle) The phase of LHNs firing advances as odor concentration increases. The FB model (red) generated an almost linear shift with increasing odor concentration. The FF model (blue) produced a drastic phase shift between low and medium concentration, which levels out to no change at high odor concentrations. (Right) GGN responds much later in the FB model than in the FB model. Results from the FB model match observations made *in vivo*.

To compare phase response properties associated with FF vs FB inhibition, we used the odor-elicited responses of KCs, LHNs, and GGN to construct circular phase diagrams [12], and then identified the mean phase responses elicited by different odor concentrations in our models. All cell populations exhibited phase locking to the LFP constructed from the population responses of PNs (see Fig 6). Both FF and FB inhibition produced similar phase dynamics in the KC population. We observed increases in the synchrony of KC firing, with more KCs firing simultaneously as odor concentration increased (Fig 6A). Thus, increases in the synchrony of the KC population could potentially provide a signal to post-synaptic neurons of olfactory system to help classify odor concentration. The phase of spikes in KCs became slightly delayed as odor concentration increased, but this shift was relatively small (Fig 6C).

LHNs also displayed similar properties of phase locking in both FF and FB conditions, with no obvious differences between models (Fig 6A). In both FF and FB models LHN spike phase advanced significantly as odor concentration increased (Fig 6C), consistent with observations made *in vivo* [12]. However, the FB model produced a fairly linear shift across concentrations, whereas the FF model produced a steep initial shift in phase followed by little to no phase shift from medium to high odor concentration (Fig 6C). The information in the linear phase shift created by FB model could offer postsynaptic cells a simple way to decode odor concentration, but such information would be more difficult to recover in the FF model.

GGN increased its extent of phase locking as odor concentration increased in both FF and FB models (Fig 6A). However, the two models produced very different phase responses in GGN (Fig 6C). The FF model produced a GGN response with no substantial phase shift with odor concentration, and a peak of activity at ~π/2 degrees. In contrast, the FB model produced a GGN response occurring much later in the oscillation cycle at ~3π/2 degrees.

Fig 6B summarizes the relative order of firing in major cell populations within each oscillatory cycle. In the FF model (Fig 6B, right), at medium concentration, PNs responded first, then LHNs, GGN, and KCs. In the FB model (Fig 6B, left), at medium concentration, PNs, KCs, and LHNs, responded within the first [π/2,π] degrees, while GGN responded at a later phase position. This sequence of phase responses was similar to that observed *in vivo* (Fig 6B, center).

**Fig 7:**
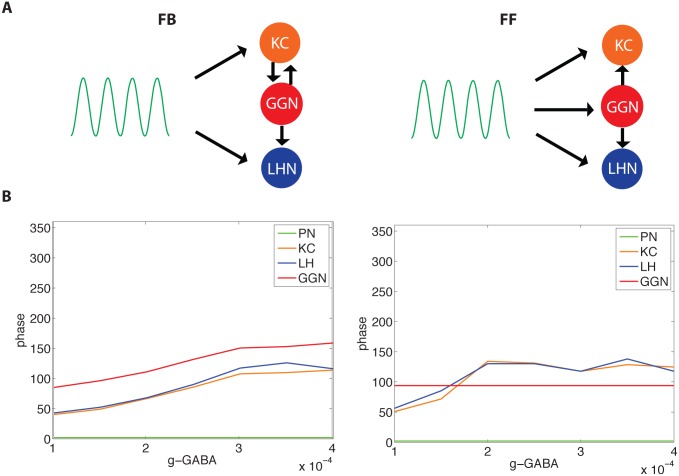
Simplified model with sine-wave input and single cells representing each cell population illustrates phase locking across a range of inhibitory synaptic strengths. Single cells: projection neuron (PN, green), Kenyon cell (KC, orange), lateral horn neuron (LHN, blue), giant GABAergic neuron (GGN, red). (A) Structure of the circuit. (B, Left) Feedback (FB) inhibitory model shows cells fired in the same order regardless of inhibitory synaptic strengths, although the KC, LHN, and GGN do fire slightly later as the inhibitory synapses were strengthened. (B, Right) Feed-forward (FF) inhibitory model: since GGN responses were determined by PN and not KCs, GGN does not phase shift. KC and LHN responses changed phase as inhibitory synaptic strength varied. KCs and LHNs fired after GGN for all values of inhibition except the weakest ones.

Our results suggested that the phase relationships among participating neurons are determined by the motif of the circuitry, FF or FB. To further explore this prediction we designed a very simplified model with a single cell representing each neural population (Fig 7A). Oscillatory AL output was simulated by a sine-wave delivered to specific neurons depending on the motif of the inhibitory circuit. Fig 7B shows the relative phase of cell firing plotted as a function of the strength of inhibition. In the FF model, PNs responded first, then GGN, with the LHN and KC firing with similar timing, except for the weakest inhibitory synaptic strength tested. In contrast, the FB circuit generated an order of spiking similar to that observed *in vivo*—KCs, LHNs, then GGN—across the entire range of inhibitory strength.

## Discussion

In ant and fly, output behavior signals are sent from the lateral protocerebrum, which contains the lateral horn [39–41]. The MB is connected to the LH through extrinsic neurons [42, 43]. Some of these extrinsic MB output neurons appear to contain odor reward information [44]. The structures within the MB, including its feedback circuits, are important for memory [16, 45, 46]. In the mammalian olfactory cortex and in honeybee MB both FF and FB inhibition exist in parallel [3, 8]. In the locust and fly, recent results provide support for FB inhibition; earlier work inconsistent with more recent results provided support through *in vivo* research and models for FF inhibition [10–12, 47].

In the locust little is known about functional roles of the LH. In the fly there is evidence that the LH processes odors that carry meaning innately [41, 48]. However, neurons examined in the locust LH respond robustly to many types of odor, ruling out a simple role in encoding innate responses [49].

Our use of classification analysis serves two functions. First, in a general sense, classification analysis provides a useful assessment of information content; by testing classification performance, we can learn about the way information is distributed in the LH and other locations in the olfactory system. Second, more specifically, classification tests allow us to assess how the information content of LHNs is affected by other parts of the olfactory system. The giant GABAergic neuron (GGN) inhibits both the KCs and LHNs. We sought to test in the model how GGN activity impacts odor representation and coding in the LH.

We developed a set of computational models of the insect olfactory system to provide a comparative analysis of the characteristics of feedforward (FF) vs. feedback (FB) inhibitory motifs. In this study we sought to determine what observations (such as sparseness of the KCs responses, phase relationships between spiking in different classes of neurons, etc) are direct consequences of the known inhibitory structure of the locust olfactory system and which are more general features that arise regardless of the nature of the inhibition. Since the basic structure of our model is rather generic (at least for insects), our model makes predictions relevant to studies of other animals where the exact nature of the inhibitory mechanisms are not yet well known.

While both models were able to maintain sparse responses in the target cell populations, the models had different efficiency profiles across ranges of concentrations: the FF model was more efficient at very low odor concentrations, and the FB model provided stronger inhibition at high concentrations. Furthermore, the two models led to distinct phase relationship profiles across cell types that could provide a tool for revealing the circuit connectivity of unknown networks. Both inhibitory motifs are ubiquitous in the insect and vertebrate brain; therefore predictions from our study of locust olfaction can be generalized to other brain circuits.

To what extent do the different response patterns of KCs and LHNs depend upon the different wirings of FF and FB inhibition? If it were possible to constrain our FF and FB models to provide identical inhibition for each odor concentration, we would likely find the responses of KCs and LHNs to be about the same. However, while the models can be adjusted to maintain the same average level of inhibition across concentrations, the different wiring patterns underlying the different inhibitory mechanisms in the FF and FB models make it impossible to keep inhibition identical across all conditions. This leads to important differences in response dynamics between the two models characterized in our study. We conclude that the wiring patterns of FB or FF inhibition generate different KC and LHN responses across a range of concentrations. Our results reported here are based on extensive testing of both models and are robust and consistent across wide ranges of parameters.

### GGN is sufficient to account for sparse representation in KCs and dense responses in LHNs

Sparse codes support learning and memory by reducing interference between memory traces, increase storage capacity, and save energy expended by action potentials [16, 18, 50, 51]. Synaptic inhibition likely provides the most powerful mechanism for sparsening neuronal representations. In many systems sparsening is achieved by a large population of the local inhibitory neurons providing either feedback or feedforward inhibition to principal neurons. As in many brain areas, (e.g., olfactory cortex [52], hippocampus [4, 53], cerebellum [54, 55], LGN [56]), feedforward inhibition mediated by a population of inhibitory cells in the locust lateral horn (LH) was until recently thought to provide the necessary inhibitory input to the Kenyon cells (KCs) of the mushroom body [9, 23]. Computer models revealed that FF inhibition could be highly efficient for maintaining the sparseness of responses in KCs across a broad range of odor concentrations [10, 11]. Models also showed that increased PN synchronization across odor concentrations could create advancing LHN spike times [57], in turn providing stronger inhibition on the KCs, counterbalancing excitation from PNs and producing a sparse KC response.

In the locust, a single giant GABAergic neuron (GGN), rather than a group of LHNs, has been shown to provide inhibition to the MB calyx [12, 19]. With extremely wide dendritic arborizations, GGN integrates input from possibly all KCs and, in turn, provides inhibition back to KCs, closing an inhibitory feedback loop. In *Drosophila* sparse coding is maintained in the KCs through feedback inhibition by a single GABAergic neuron, the anterior paired lateral (APL) neuron. The activation of this neuron was shown to enhance odor memory [13]. One could predict that GGN in the locust would similarly increase the specificity of olfactory memory.

We found that both FF and FB inhibitory motifs generated sparse codes, supporting previous work showing both motifs increase KC excitatory current threshold to compensate for changing input intensity elicited by changing odor concentrations [1, 3, 7, 11, 52, 59]. Roughly 10% of KCs respond to any given odor by spiking 1–3 times [9]. The FB inhibition model provided a better match for observations made *in vivo*: using the middle range of the odor concentrations, 11.5% of modeled KCs responded with an average of 2.6 action potentials each, compared to the FF model, with 8.5% active KCs, each firing an average of 3.8 action potentials. The FF inhibition model responded to stimuli with fewer cells firing more action potentials compared to the FB inhibitory motif. Although the type of inhibitory motif affected overall activity, GGN was sufficient to create KC sparse olfactory responses in both models.

GGN—mediated inhibition affects both the KCs and LHNs, although both classes of neurons respond differently to odor presentations. All types of LHNs (classes 1–4) respond to each odor with dense spiking [12]. Regardless of inhibitory motif, all odor conditions activated all modeled LHNs. *In vivo*, the LH activity dynamics may be more complex than explored here; GABAergic neurons within the LH may also inhibit the LHNs [12]. The coding strategy used by LHNs remains an open question; in locusts these neurons do not provide labeled lines for specific odors, and do not respond only to innately meaningful odors. Multimodal cells in the lateral horn, which process visual and olfactory information, may not receive direct input from PNs. LHNs implemented in our model (C1-C4 [12]) may provide excitatory input to these specialized multimodal cells. Since the mushroom bodies serve important memory functions in insects, one can speculate the KCs->GGN->LHNs pathway is utilized to modulate responses depending on memory context. It is not clear how odor-specific or memory-specific control can be achieved if a single cell, GGN, is responsible. An inhibitory pathway from the MB to the LHNs may exist involving beta-lobe neurons [16], but the exact anatomical organization of these circuits has yet to be determined.

In the locust, the KCs excite extrinsic MB neurons, some of which have been shown to have spike timing dependent plasticity (STDP) at the synapse from the KCs to the extrinsic neurons [16, 58]. Nowotny et al (2005) [11] tested the idea that STDP could occur at synapses connecting KCs to their post-synaptic neurons, and found, through models, that FF inhibition on to the KCs could create enough gain control for these STDP synapses to function effectively. Furthermore, they found that the gain control provided by FF inhibition helps a little when discriminating very different odors, but becomes critical when discriminating very similar odors.

### Inhibitory motif impacted odor representation and classification error

Insects can make decisions in olfactory tasks within 300–500 ms of the start of an odor presentation [60]. Physiological results show the initial part of an odor presentation is sufficient for successful odor classification [61], and every part of the odor response contains enough information for successful classification [62]. Both inhibitory models achieved successful classification performance (classification error less than 10%) within the first 250–300 ms of odor presentation for certain conditions. The FF inhibition model successfully classified KC odor responses, except for similar odors of low concentrations; LHNs had difficulty with similar odors. The FB inhibition model successfully classified responses to different odors, and LHNs provided generally better classification performance in the FB model. *In vivo*, it is difficult to measure KC classification success owing to the large numbers of cells and their sparse activity. In locusts, responses of single LHNs can be used to classify 60–95% of odors within 250–300 ms, timing [12], consistent with our models’ predictions. Population measures from LHNs, regardless of the type of inhibitory motif, performed poorly classifying similar odors. Anatomical evidence suggests both FF and FB inhibition act on the LHNs *in vivo* [12]. Some of the difficulty our models faced with odor classification in LHNs might be resolved by including additional inhibitory interactions that are not yet well characterized.

### Inhibitory motif determines the relative timing of response

In both inhibitory motif models, spiking in LHNs advanced in phase relative to the field potential as odor concentration increased, consistent with experiments performed *in vivo* [12]. Oscillations are faithfully transmitted throughout the olfactory system [58], suggesting this shift in phase could provide downstream neurons information about odor concentration [12, 63]. The FB inhibition model created a more linear shift in phase across concentrations than the FF model, potentially advantageous for communicating information about odor concentration to post-synaptic neurons [63].

Systematic changes in the spike timing of LHNs could affect the integration of olfactory and visual information by downstream multimodal neurons [12]. When odor concentrations are low, action potentials in LHNs are less synchronized, potentially allowing downstream neurons to favor visual input. When odor concentrations increase, LHNs increasingly phase lock and provide synchronized output, and thus may be more likely to elicit action potentials in multimodal follower cells regardless of visual input.

Both models revealed small phase shifts in KC spiking as odor concentration changed. *In vivo* recordings show only small or zero phase shifts in these neurons; however, the sparseness of odor-elicited spiking in KCs makes it difficult to reliably estimate the mean response phase. KCs make excitatory projections to a relatively small population of type 2 β-lobe neurons (bLN-2) [49]. This pathway is plastic in response to experience, thus supporting learning [13, 16, 58, 64–66]. Since plasticity of the KCs-> bLN-2 pathway is based on spike timing [16], the stability of spike timing in KCs may support reliable synaptic changes and communication between the KCs and bLN-2s [63, 64]

The FB inhibitory motif produced spike phase responses matching those observed *in vivo*. Similarly, the FB model yielded a sequence of spiking, PNs-KCs-LHNs-GGN, also matching results obtained *in vivo* [9, 12], whereas the FF model generated a different spike order: PNs-LHNs-GGN-KCs. We confirmed this finding with a simplified model for a range of the inhibitory synaptic strength.

## Conclusion

FF and FB inhibitory motifs exhibited similar cellular population behaviors. Models featuring either motif maintained the sparseness of odor-elicited spiking in KCs, but with different activity patterns; in the FF model, fewer KCs responded to each odor presentation with more spikes; in the FB model, more KCs responded to each odor with fewer spikes. Both inhibitory motifs provided similar performance in odor discrimination. The FB inhibitory motif produced a more nimble GGN response, with a wider range of cellular activity and inhibition on post-synaptic cells compared to the GGN response shaped by the FF inhibitory motif. Only the FB inhibitory motif created a phase response consistent with results recorded *in vivo*. These findings provide a general view of the attributes of FF and FB inhibition, and, in the specific case of olfaction in the insect, provide evidence that FB inhibition controls the responses of mushroom body neurons.
